# Genetic Variability of the Vitamin D Receptor Affects Susceptibility to Parkinson’s Disease and Dopaminergic Treatment Adverse Events

**DOI:** 10.3389/fnagi.2022.853277

**Published:** 2022-04-19

**Authors:** Sara Redenšek, Tilen Kristanc, Tanja Blagus, Maja Trošt, Vita Dolžan

**Affiliations:** ^1^Pharmacogenetics Laboratory, Institute of Biochemistry and Molecular Genetics, Faculty of Medicine, University of Ljubljana, Ljubljana, Slovenia; ^2^Department of Neurology, University Medical Centre Ljubljana, Ljubljana, Slovenia

**Keywords:** vitamin D receptor, Parkinson’s disease, biomarker, risk, susceptibility, adverse events, vitamin D, polymorphism

## Abstract

Vitamin D is a lipid-soluble molecule and an important transcriptional regulator in many tissues and organs, including the brain. Its role has been demonstrated also in Parkinson’s disease (PD) pathogenesis. Vitamin D receptor (VDR) is responsible for the initiation of vitamin D signaling cascade. The aim of this study was to assess the associations of *VDR* genetic variability with PD risk and different PD-related phenotypes. We genotyped 231 well characterized PD patients and 161 healthy blood donors for six *VDR* single nucleotide polymorphisms, namely rs739837, rs4516035, rs11568820, rs731236, rs2228570, and rs1544410. We observed that *VDR* rs2228570 is associated with PD risk (*p* < 0.001). Additionally, we observed associations of specific *VDR* genotypes with adverse events of dopaminergic treatment. *VDR* rs1544410 (GG vs. GA + AA: *p* = 0.005; GG vs. GA: *p* = 0.009) was associated with the occurrence of visual hallucinations and *VDR* rs739837 (TT vs. GG: *p* = 0.036), rs731236 (TT vs. TC + CC: *p* = 0.011; TT vs. TC: *p* = 0.028; TT vs. CC: *p* = 0.035), and rs1544410 (GG vs. GA: *p* = 0.014) with the occurrence of orthostatic hypotension. We believe that the reported study may support personalized approach to PD treatment, especially in terms of monitoring vitamin D level and vitamin D supplementation in patients with high risk *VDR* genotypes.

## Introduction

Vitamin D is a lipid-soluble secosteroid obtained through dermal synthesis by exposure to sunlight or through dietary intake (Fullard and Duda, 2020). The active compound acts as a hormone and binds to the vitamin D receptor (VDR), which regulates expression of more than 200 different genes (Samuel and Sitrin, 2008; Fullard and Duda, 2020; Lv et al., 2020). VDR is present in several brain areas, such as hippocampus, hypothalamus, thalamus, cortex, and substantia nigra (Moretti et al., 2018). Vitamin D thus has an important role in the central nervous system as it is involved in neurotransmission, neuroprotection, and neuroplasticity (DeLuca et al., 2013). All this prompted many studies to investigate the relation between vitamin D homeostasis and neurological disorders. Several disorders with diverse etiologies have thus been associated with vitamin D levels already: multiple sclerosis, Alzheimer’s disease, amyotrophic lateral sclerosis, and Parkinson’s disease (PD) (DeLuca et al., 2013).

Parkinson’s disease is the second most prevalent neurodegenerative brain disorder and is presenting with a variety of motor and non-motor signs and symptoms. Its hallmarks are aggregation of the misfolded α-synuclein in the intracellular inclusions called Lewy bodies and degeneration of dopaminergic neurons in the nigrostriatal pathway (Kalia and Lang, 2015; Poewe et al., 2017; Tolosa et al., 2021). In later stages of PD, motor and non-motor complications due to prolonged dopaminergic treatment develop and they severely impact patients’ and caregivers’ health related quality of life (Zhao et al., 2021). Vitamin D deficiency is common in PD patients. However, it is still unknown how is it related to PD, whether it is a cause or a consequence of PD pathology (Luo et al., 2018). Nevertheless, it was shown in rodent models of PD that VDR affects expression of tyrosine hydroxylase as the rate-limiting step in dopamine synthesis, and also the expression of glial cell line-derived neurotrophic factor (GDNF), which facilitates neuronal regrowth and protects dopaminergic nerve terminals (Lv et al., 2020).

Studies have already shown associations between vitamin D levels and PD risk. A 29-year long prospective study conducted in Finland showed that people with increased vitamin D serum concentrations had lower risk for PD development (Knekt et al., 2010). Several studies also showed that higher exposure to sunlight, which increases vitamin D production in the skin, protects against PD development (Kenborg et al., 2011; Zhu et al., 2014; Wang et al., 2016; Kravietz et al., 2017). Additionally, vitamin D levels were also shown to be correlated with severity of motor symptoms in PD, scored by the MDS-Unified Parkinson’s Disease Rating Scale (MDS-UPDRS) and Hoehn and Yahr (H&Y) scales. Both scales were inversely correlated with serum and plasma vitamin D levels (Chitsaz et al., 2013; Ding et al., 2013; Fullard and Duda, 2020). There are also some reports available stating that vitamin D levels are associated with non-motor symptoms, although these studies are fairly inconsistent. Higher plasma vitamin D levels were associated with better verbal fluency and verbal memory in PD without dementia (Peterson et al., 2013). Several studies have shown an association between vitamin D levels and depression in the general elderly population (Wilkins et al., 2006; Peterson et al., 2013), similarly as orthostatic hypotension (Duval et al., 2015). Finally, vitamin D levels also showed association with olfactory dysfunction (Kim et al., 2018).

Genetic variability in *VDR* has already been investigated in several different settings. More specifically, *VDR* rs2228570 was associated with the risk of PD development (Han et al., 2012; Török et al., 2013; Li et al., 2015; Niu et al., 2015; Wang et al., 2019; Gao et al., 2020; Hu et al., 2020; Agliardi et al., 2021). This single nucleotide polymorphism (SNP) was also associated with the disease progression (Suzuki et al., 2012) and with cognitive decline in PD patients (Gatto et al., 2015). A few other studies demonstrated the association of *VDR* rs1544410 with PD risk (Kim et al., 2005; Li et al., 2015).

However, none of the studies investigated the association of *VDR* genetic variability with the occurrence of adverse events (AEs) of treatment with levodopa and dopamine agonists (ADs). Additionally, no studies have assessed whether *VDR* genetic variability influences the daily dose requirements of dopaminergic treatment necessary to adequately control PD symptoms in an individual patient. The aim of our study was to address these two scientific questions and additionally also to assess the association between *VDR* SNPs and PD risk in a Slovenian cohort of PD patients.

## Materials and Methods

### Participants and Clinical Data

In total, 231 consecutive PD patients were included in this study. Patients were recruited at the Department of Neurology, University Medical Centre Ljubljana, Slovenia, between October 2016 and April 2018 (Redenšek et al., 2019). Inclusion criteria are listed elsewhere already (Redenšek et al., 2019). The criteria were the following: (1) diagnosis of PD as defined in the United Kingdom Parkinson’s Disease Society Brain Bank criteria (Goetz et al., 2008) by an experienced movement disorders specialist, (2) available clinical data, (3) at least 3 months of levodopa and/or DAs treatment duration, (4) ongoing dopaminergic treatment with levodopa and/or DAs. Patients with atypical and secondary forms of parkinsonisms were not included in the study.

Patients and their caregivers underwent a structured interview to obtain demographic and clinical data of interest. Additional clinical data was obtained from medical records. Data on gender, side of disease initiation, presence of tremor, treatment with DAs, age at diagnosis, disease duration, and levodopa treatment duration was collected. Levodopa equivalent dose (LED) at the inclusion in the study was used as a measure of the disease severity level. We also collected data on the occurrence of AEs during the course of dopaminergic treatment. The dopaminergic treatment related AEs of interest were the following: motor fluctuations (MFs), dyskinesia, excessive daytime sleepiness and sleep attacks, visual hallucinations (VHs), nausea/vomiting, orthostatic hypotension, peripheral edema, and impulse control disorders (ICDs).

A control group of 161 unrelated Slovenian blood donors without neurological and oncological disorders were included in the study for the purpose of PD susceptibility evaluation. We collected data on their age and gender.

The study protocol was approved by the Slovenian Ethics Committee for Research in Medicine (KME 42/05/16). All subjects gave written informed consent in accordance with the Declaration of Helsinki.

### Single Nucleotide Polymorphism Selection

In total, six functional SNPs from regulatory regions of the *VDR* gene were selected for the analysis, namely rs739837, rs4516035, rs11568820, rs731236, rs2228570, and rs1544410. All of the SNPs had a function already reported in the literature or predicted by the SNP function prediction tool (Xu and Taylor, 2009). We selected SNPs based on their previous association with PD and related phenotypes (Kim et al., 2005; Han et al., 2012; Suzuki et al., 2012; Török et al., 2013; Gatto et al., 2015; Li et al., 2015; Niu et al., 2015; Wang et al., 2019; Gao et al., 2020; Hu et al., 2020; Agliardi et al., 2021) and based on their location in the gene and minor allele frequency (MAF). Only very common SNPs with the MAF above 10% were selected. SNPs from coding region, 5′UTR, and 3′UTR were prioritized. Two selected SNPs are located in the coding region (rs731236 and rs2228570), two SNPs in the 5′UTR (rs4516035 and rs11568820), one in the 3′UTR (rs739837), and one in the intronic region (rs1544410). The intronic SNP was included into the investigation due to the previously reported association with PD risk (Kim et al., 2005; Li et al., 2015). The characteristics of the investigated SNPs are presented in the Table 1.

**TABLE 1:** Genotype frequencies and characteristics of the genetic variants studied in our PD cohort.

Gene	Polymorphism	Synonym	Location in gene	MAF (%)	Predicted SNP function*	Genotype	*N* (%) patient cohort	*N* (%) control cohort	HWE equilibrium *p*-value (control cohort)
*VDR*	rs739837 c.*308C>A	*Bgl*I	3′UTR	44.63	Affects miRNA binding	TT	66(28.6)	43(26.7)	0.819
						TG	112(48.5)	79(49.1)	
						GG	53(22.9)	39(24.2)	
	rs4516035 c.-1413A>G	A-1012G	5′UTR	42.25	Affects transcription factor binding	TT	63(27.3)	40(24.8)	0.169
						CT	117(50.6)	89(31.7)	
						CC	51(22.1)	32(19.9)	
	rs11568820 g.1270G>A	Cdx2	5′UTR	22.76	Affects transcription factor binding	GG	159(68.8)	108(67.1)	0.133
						GA	64(27.7)	51(31.7)	
						AA	8(3.5)	2(1.2)	
	rs731236 p.Ile352=	*Taq*I	Coding region	39.96	Affects splicing	TT	84(36.4)	60(37.3)	0.367
						TC	113(48.9)	72(44.7)	
						CC	34(14.7)	29(18.0)	
	rs2228570 p.Met51Thr	*Fok*I	Coding region	37.77	Non-synonymous Affects splicing	CC	88(38.1)	60(37.3)	0.117
						TC	102(44.2)	84(52.2)	
						TT	41(17.7)	17(10.6)	
	rs1544410** c.1174+283G>A	*Bsm*I	Intron	40.36	Alters mRNA stability	GG	78(33.8)	58(36.0)	0.365
						GA	119(51.5)	72(44.7)	
						AA	34(14.7)	30(18.6)	

*SNP function predicted by the tool (Xu and Taylor, 2009). **Genotype data is missing for one control sample. MAF, minor allele frequency; HWE, Hardy–Weinberg equilibrium.

### DNA Isolation and Genotyping

Peripheral blood samples were obtained for DNA extraction and genomic DNA was isolated using the FlexiGene DNA Kit (Qiagen, Hilden, Germany) in the course of our previous study (Redenšek et al., 2019). Six SNPs (*VDR* rs739837, rs4516035, rs11568820, rs731236, rs2228570, and rs1544410) were genotyped with KASPar assays (KBiosciences, Herts, United Kingdom and LGC Genomics, United Kingdom) according to manufacturer’s instructions. In total, 10% of samples were genotyped in duplicate as quality control and all the results were concordant.

### Statistical Analysis

Median and 25th to 75th percentile range were used to describe central tendency and variability of continuous variables, while frequencies were used to describe the distribution of categorical variables. The agreement of genotype frequencies with Hardy–Weinberg equilibrium was assessed by Chi-squared test in the control cohort.

Logistic regression was used to calculate odds ratios (ORs) and 95% confidence intervals (95% CIs) to examine the associations of selected SNPs and clinical data with the risk for AEs. Logistic regression was used for the risk analysis as well. Dominant, recessive, and additive genetic models were used according to the genotype frequencies. Non-parametric Mann−Whitney U test and Kruskal−Wallis test were used to assess the effects of genotypes on the LED. ANCOVA was used to adjust for the clinical variables.

All statistical tests were two-sided. Bonferroni correction was used to account for multiple comparisons to prevent false positive results. *p*-values up to 0.0083 (0.05/6) were considered statistically significant, while *p*-values between 0.0083 and 0.0500 were considered nominally significant. For an allelic variant with an average MAF of 0.39 and with a 32% prevalence of an AE, this study had 80% or more power to detect OR of 0.40 or less and OR of 2.23 or more. More detailed power calculations are presented in the Supplementary Table 1. Power calculations were conducted by the PS Power and sample size calculations, version 3.0. All statistical analyses were carried out by IBM SPSS Statistics, version 21.0 (IBM Corporation, Armonk, NY, United States).

## Results

### Patients’ Characteristics

A total of 132 male and 99 female PD patients were included in the study. Patients’ median age at enrollment was 72.5 years (65.7–78.0). Their median age at diagnosis was 62.1 years (54.8–71.7) and their median disease duration was 7.6 years (3.8–13.6). The median dopaminergic treatment duration was 7.3 years (3.6–13.5) and the median LED at enrollment was 975 mg/day (600–1,363.5).

The control cohort included 125 men and 36 women with a median age of 55 years (52–58.5).

The main patient and control cohort characteristics are presented in the Table 2. Other characteristics of the patient cohort and the data on the frequencies of AEs are summarized in the Supplementary Table 2, which is adjusted according to Redenšek et al. (2019).

**TABLE 2:** Patient and control cohort characteristics.

Characteristic		Patient cohort (*N* = 231)	Control cohort (*N* = 161)
Gender	Male (%)	132 (57.1)	125 (77.6)
	Female (%)	99 (42.9)	36 (22.4)
Tremor-predominant PD	No (%)	46 (19.9)	
	Yes (%)	185 (81.1)	
Age at enrollment	Median (25–75%), years	72.5 (65.7–78.0)	55 (52–58.5)
Age at diagnosis	Median (25–75%), years	62.1 (54.8–71.7)	
Disease duration	Median (25–75%), years	7.6 (3.8–13.6)	
LED at enrollment^*,^**	Median (25–75%), mg/day	975 (600–1,363.5)	

*LED calculated according to Tomlinson et al. (2010). **Data missing for four patients.

### Single Nucleotide Polymorphism Genotyping Analysis

In total, six SNPs were analyzed. Data on genotype frequencies of these variants along with their locations, MAF, and predicted functions are presented in the Table 1. None of the genotype distributions deviated from the Hardy–Weinberg equilibrium as seen in Table 1.

### Influence of Genetic Variability on the Risk for Parkinson’s Disease Development

Risk analysis showed a significant association between *VDR* rs2228570 and PD development. Carriers of at least one *VDR* rs2228570 T allele had increased odds for development of the disease (OR = 2.75; 95% CI = 1.73–4.38: *p* < 0.001). The association remained significant after adjustment for age and gender (OR = 3.07; 95% CI = 1.86–5.08: *p* < 0.001). The other investigated polymorphisms were not associated with PD risk (Table 3).

**TABLE 3:** Associations between *VDR* genotypes and the risk for Parkinson’s disease.

Gene	Polymorphism	Genotype	Parkinson’s disease			Parkinson’s disease*		
			OR	95% CI	*p*-value	OR	95% CI	*p*-value
*VDR*	rs739837	TT	Ref.					
		TG	0.92	0.57–1.49	0.746	0.95	0.56–1.59	0.836
		GG	0.89	0.50–1.56	0.672	0.86	0.46–1.59	0.626
		TG + GG	0.91	0.58–1.43	0.685	0.92	0.56–1.50	0.732
	rs4516035	TT	Ref.					
		CT	0.84	0.52–1.35	0.463	0.98	0.58–1.66	0.947
		CC	1.01	0.56–1.83	0.969	1.17	0.62–2.23	0.627
		CT + CC	0.88	0.56–1.40	0.591	1.03	0.63–1.70	0.900
	rs11568820	GG	Ref.					
		GA	0.85	0.55–1.33	0.478	0.80	0.50–1.29	0.363
		AA	2.72	0.57–13.04	0.212	2.19	0.42–11.49	0.356
		GA + AA	0.92	0.60–1.42	0.715	0.86	0.54–1.36	0.512
	rs731236	TT	Ref.					
		TC	1.12	0.72–1.75	0.614	1.16	0.72–1.87	0.544
		CC	0.84	0.46–1.52	0.560	0.97	0.51–1.85	0.916
		TC + CC	1.04	0.69–1.58	0.855	1.11	0.71–1.74	0.658
	rs2228570	CC	Ref.					
		TC	0.83	0.53–1.28	0.397	0.92	0.58–1.48	0.740
		TT	1.64	0.86–3.16	0.136	1.63	0.81–3.29	0.169
		TC + TT	**2.75**	**1.73–4.38**	**<0.001**	**3.07**	**1.86–5.08**	**<0.001**
	rs1544410	GG	Ref.					
		GA	1.23	0.79–1.92	0.368	1.25	0.77–2.03	0.360
		AA	0.84	0.46–1.53	0.574	0.94	0.49–1.79	0.841
		GA + AA	1.12	0.73–1.70	0.612	1.16	0.74–1.84	0.516

*Adjusted for gender and age. Statistically significant results are printed in bold text.

### Influence of *VDR* Genetic Variability on the Symptomatology of Parkinson’s Disease

LED was used as a measure of PD severity at the point of enrollment. The *VDR* rs4516035 was nominally significantly associated with the dose of dopaminergic drugs needed to adequately manage PD symptoms. Carriers of at least one *VDR* rs4516035 C allele required higher LED than carriers of the TT genotype (*p* = 0.023). There were no significant or nominally significant associations detected in the additive model. After adjustment of the nominally significant association from the dominant model for gender and disease duration, only disease duration showed significant effect on the LED (*p* < 0.001). Complete data is presented in the Supplementary Table 3.

### Influence of *VDR* Genetic Variability on the Occurrence of Adverse Events of Dopaminergic Treatment

We checked the association of selected SNPs with the occurrence of eight different AEs of dopaminergic treatment. We found significant and nominally significant associations with four AEs, namely VHs, orthostatic hypotension, peripheral edema, and MFs.

Carriers of the *VDR* rs1544410 GA genotype (OR = 0.43; 95% CI = 0.22–0.84; *p* = 0.013) or carriers of at least one *VDR* rs1544410 A allele (OR = 0.46; 95% CI = 0.25–0.86; *p* = 0.014) had decreased odds for development of VHs. In regards to orthostatic hypotension, carriers of the *VDR* rs739837 GG genotype had increased odds (OR = 2.23; 95% CI = 1.05–4.70; *p* = 0.036) for development of this AE, whereas carriers of the *VDR* rs731236 TC (OR = 0.52; 95% CI = 0.29–0.93; *p* = 0.026) and CC genotype (OR = 0.38; 95% CI = 0.16–0.91; *p* = 0.029), as well as carriers of at least one *VDR* rs731236 C allele (OR = 0.48; 95% CI = 0.28–0.84; *p* = 0.010) had decreased odds for development of orthostatic hypotension. *VDR* rs1544410 was also associated with orthostatic hypotension. Carriers of the *VDR* rs1544410 GA genotype (OR = 0.48; 95% CI = 0.26–0.86; *p* = 0.013), AA genotype (OR = 0.42; 95% CI = 0.18–0.99; *p* = 0.046), and carriers of at least one A allele (OR = 0.46; 95% CI = 0.26–0.81; *p* = 0.007) had decreased odds for development of this AE. Finally, carriers of the *VDR* rs731236 TC genotype had decreased odds for development of peripheral edema (OR = 0.43; 95% CI = 0.20–0.90; *p* = 0.026). The results for all the investigated SNPs and non-motor AEs are presented in Supplementary Tables 4–9.

In regards to the motor AEs, only MFs presented with a nominally significant association with the *VDR* genetic variability. Carriers of at least one *VDR* rs2228570 T allele had increased odds for development of MFs (OR = 2.01; 95% CI = 1.01–4.02; *p* = 0.047). All the data for motor AEs is presented in Supplementary Tables 10, 11.

Significant and nominally significant associations obtained in univariate analysis were then adjusted for significant clinical parameters in multivariate analysis. Clinical parameters associated with the occurrence of non-motor and motor AEs in our patient cohort were already reported in our previous study (Redenšek et al., 2019). These significant clinical parameters were used for adjustments of associations with the corresponding AEs. The results of the multivariate analysis are presented in the Table 4. All of the nominally significant or significant associations from the univariate analysis retained significance levels also after adjustments, except for the association of the *VDR* rs2228570 with MFs. Additionally, the association between *VDR* rs731236 and peripheral edema was not adjusted for any clinical parameter because none of them were significantly associated with this AE. Both associations between the occurrence of VHs and the *VDR* rs1544410 genotype remained significant with *p*-values dropping even lower compared to the univariate analysis. Additionally, the majority of associations between *VDR* genetic variability and the occurrence of orthostatic hypotension remained nominally significant. Carriers of the *VDR* rs739837 GG genotype (OR = 2.23; 95% CI = 1.05–4.71; *p* = 0.036) had increased odds for development of this AE. Carriers of the *VDR* rs731236 TC genotype (OR = 0.52; 95% CI = 0.29–0.93; *p* = 0.028), CC genotype (OR = 0.39; 95% CI = 0.16–0.94; *p* = 0.035), or at least one C allele (OR = 0.49; 95% CI = 0.28–0.85; *p* = 0.011) had decreased odds for development of orthostatic hypotension. Finally, carriers of the *VDR* rs1544410 GA genotype (OR = 0.48; 95% CI = 0.27–0.86; *p* = 0.014) had decreased chances for this AE. The remaining two associations showed *p*-values above 0.05.

**TABLE 4:** Significant associations from the univariate analysis adjusted for significant clinical parameters.

SNP	Adverse event	Adjusted for	Genotype	OR adj.*	95% CI	*p*-value
*VDR* rs1544410	Visual hallucinations	Age at diagnosis	GG	Ref.		
			**GA**	**0.41**	**0.21–0.80**	**0.009**
			AA	0.51	0.20–1.31	0.159
			**GA + AA**	**0.96**	**0.94–0.99**	**0.005**
*VDR* rs739837	Orthostatic hypotension	Age at diagnosis	TT	Ref.		
			TG	1.17	0.61–2.23	0.645
			**GG**	**2.23**	**1.05–4.71**	**0.036**
			TG + GG	1.45	0.79–2.65	0.232
*VDR* rs731236	Orthostatic hypotension	Age at diagnosis	TT	Ref.		
			**TC**	**0.52**	**0.29–0.93**	**0.028**
			**CC**	**0.39**	**0.16–0.94**	**0.035**
			**TC + CC**	**0.49**	**0.28–0.85**	**0.011**
*VDR* rs1544410	Orthostatic hypotension	Age at diagnosis	GG	Ref.		
			**GA**	**0.48**	**0.27–0.86**	**0.014**
			AA	0.43	0.18–1.02	0.054
			GA + AA	1.01	0.99–1.03	0.407
*VDR* rs731236	Peripheral edema	*No clinical parameter was significantly associated with this adverse event*	TT	Ref.		
			TC			
			CC			
			TC + CC			
*VDR* rs2228570	Motor fluctuations	Side of disease initiation Tremor-predominant PD Ever being treated with DAs Age at diagnosis	CC	Ref.		
			TC	1.41	0.69–2.92	0.348
			TT	0.57	0.23–1.39	0.217
			TC + TT	2.12	0.94–4.76	0.070

*OR values from univariate analysis adjusted for clinical parameters from the third column. Statistically significant results are printed in bold text.

## Discussion

The presented study assessed the associations between selected functional SNPs of *VDR* and symptomatology of PD. This is the first study to report strong associations between *VDR* polymorphisms and non-motor AEs of dopaminergic treatment, such as VHs and orthostatic hypotension. Additionally, it confirms the association of *VDR* rs2228570 with the risk of PD development. However, we observed no statistically significant associations with LED and consequently disease severity.

In our cohort, *VDR* rs2228570 conferred almost threefold increase in the risk for PD development in carriers of at least one T allele. The product of *VDR* rs2228570 wild-type C allele is a three amino-acids shorter version of the protein and is more transcriptionally potent than the longer version (Uitterlinden et al., 2004; Lv et al., 2020). This is even more important since VDR regulates the expression of several neurotrophic factors, such as nerve growth factor (Wion et al., 1991), neurotrophin 3 (Neveu et al., 1994), and GDNFs (Naveilhan et al., 1993). It has also been reported that *VDR* rs2228570 C allele carriers have higher capacity for intestinal calcium absorption, which may lead to higher vitamin D levels (Arai et al., 1997; Uitterlinden et al., 2004). Additionally, vitamin D signaling *via* VDR is involved in protection of neurons against oxidative stress (Fullard and Duda, 2020). Since lower vitamin D levels were reported as a risk factor for PD development several times (Knekt et al., 2010), we can expect that higher vitamin D levels could present a protective influence. Similarly, it has been shown that a significantly lower (50%) dose of 1,25-dihydroxyvitamin D_3_ is necessary for the effect in the *VDR* rs2228570 CC genotype than in the *VDR* rs2228570 CT genotype carriers (Colin et al., 2000). A randomized control trial showed that vitamin D_3_ supplementation may slow the progression of PD in patients with *VDR* rs2228570 CT and TT genotypes (Suzuki et al., 2013). The TT genotype was already associated with the more severe form of PD (Suzuki et al., 2012), with cognitive decline in PD (Gatto et al., 2015), and with PD risk (Hu et al., 2020; Agliardi et al., 2021). Some reported studies also showed an opposite effect in regards to PD risk, however most of these studies were done in Asian patient cohorts (Li et al., 2015; Wang et al., 2019), which may be related to the ethnic differences in genetic predisposition to diseases. All of the above indicates and supports a very high importance of the finding that *VDR* rs2228570 genotype partly defines the development and course of PD.

We observed an association between the *VDR* rs4516035 and LED as a measure of the general disease severity, which lost its statistical significance after adjustment for the disease duration. This variant modifies recognition sequence of the GATA-3 transcription factor (Fibla and Caruz, 2010), which might decrease expression of VDR and thus lead to worse PD symptomatology and consequently higher LED. However, we can conclude that disease duration is a much more important parameter in determining LED than *VDR* rs4516035 genotype, which is in concordance with the expectations. We have to emphasize that LED is not the most suitable parameter for assessment of PD severity in a particular point of the disease course. However, PD clinical rating scales were not available at the point of patient enrollment. Nevertheless, *VDR* genotype has been assessed in association with different PD-related phenotypes already (Suzuki et al., 2012; Gatto et al., 2015), which indicates that further research in independent patient cohorts is warranted.

We also detected some interesting novel associations of *VDR* SNPs with the occurrence of AEs of dopaminergic treatment. We observed that carriers of the *VDR* rs1544410 GA genotype and carriers of at least one A allele had decreased odds for development of VHs. It has been reported that the G allele is associated with lower mRNA stability, which indicates that there could be less VDR available for vitamin D signaling (Fibla and Caruz, 2010). Knowing this, higher VDR expression might protect against VHs. Additionally, knowing the genotype of patients in advance could enable compensation for lower VDR availability with vitamin D supplementation to avoid VHs development. For example, it has already been shown in Alzheimer’s disease that vitamin D supplementation protects against psychosis (Fan et al., 2020). Furthermore, *VDR* rs739837, rs731236, and rs1544410 genotypes were strongly associated with the occurrence of orthostatic hypotension. A study by Jang et al. (2015) showed that serum 25-hydroxyvitamin D_3_ levels were significantly lower in PD patients with orthostatic hypotension in comparison to the patients without it, indicating an importance of vitamin D signaling in orthostatic hypotension occurrence. The exact mechanism is not known, but it was shown that vitamin D signaling plays a role in vascular endothelial function, blood pressure, renal function and cardiac autonomic activity (Soysal et al., 2014). We report that carriers of the *VDR* rs739837 GG genotype have more than twofold higher odds for development of orthostatic hypotension in comparison to the TT genotype. It was postulated that GG genotype leads to lower expression of VDR (Küchler et al., 2021), which might explain the increased risk for orthostatic hypotension. Furthermore, carriers of at least one *VDR* rs731236 C allele and *VDR* rs1544410 GA genotype had decreased odds for the development of orthostatic hypotension. Both genetic changes increase mRNA stability (Fibla and Caruz, 2010), which might be the reason for protective effect of the variants. Similarly, carriers of the *VDR* rs731236 TC genotype had decreased odds for the development of peripheral edema. The presented study is to the best of our knowledge the first one to report the association between *VDR* genetic variability and peripheral edema. However, the mechanism remains to be elucidated. The associations shown in this study all present with a clinical potential of pre-emptive identification of patients at risk for AE development and of patients that might benefit from vitamin D supplementation.

Our study presents important novel findings, but it should be emphasized that there are some limitations that we have to bear in mind when interpreting our results. A study cohort is of moderate size, but still comparable to other pharmacogenetic studies performed in the cohorts of PD patients and of a uniform genetic background as well. As explained above, LED is not the most suitable measure of disease severity. Clinical scales would be more relevant for this assessment. Unfortunately, this data was not available at the point of patient enrollment. The control group within the PD susceptibility analysis is in average almost 20 years younger than the patient group, which means that we might have missed some PD cases. However, the prevalence of PD is rather low in the general population, which is why we speculate that the age difference between the groups does not affect the results to a significant extent. Finally, we only considered the AEs as binary categorical variables since clinical scales are not routinely used to assess AEs in PD. Quantified severity of AEs would likely give us an opportunity to analyze associations more in depth.

Despite its limitations, the presented study provided us with important findings, which should nevertheless be assessed in independent populations as well. The association between *VDR* genetic variability and occurrence of orthostatic hypotension is a highly relevant finding which has not been elucidated yet. This indicates that patients with a specific *VDR* genotype might benefit from vitamin D supplementation to prevent orthostatic hypotension. *VDR* genotype could serve as a valuable predictive biomarker of this AE as well. Furthermore, we confirmed a very strong association between *VDR* rs2228570 and increased risk for PD development. This has recently been pointed out by several studies conducted in the Caucasian population from which a cohort of Slovenian patients included in the reported study originate as well. The latter confirms the reliability of this finding, which should be accounted for in future polygenic risk scores for prediction of PD since none of them so far take into account the *VDR* genotype (Ibanez et al., 2017; Dehestani et al., 2021). Additionally, patients with at risk *VDR* genotypes are suitable candidates for close monitoring of vitamin D levels.

## Conclusion

This is the first study that evaluated a comprehensive spectrum of PD-related phenotypes in one cohort of PD patients in relation to *VDR* genetic variability. Our results may help identify people at risk to develop PD as well as PD patients at risk to develop certain AEs of dopaminergic treatment that would benefit from close monitoring of vitamin D levels and vitamin D supplementation. Results of the present study should be validated in an independent cohort. Nevertheless, we believe that these results will contribute to a more personalized treatment strategy in individual PD patients.

## Data Availability Statement

The original contributions presented in the study are included in the article/Supplementary Material; further inquiries can be directed to the corresponding author/s.

## Ethics Statement

The studies involving human participants were reviewed and approved by the Medical Ethics Committee of the Republic of Slovenia. The patients/participants provided their written informed consent to participate in this study.

## Author Contributions

SR, MT, and VD: conceptualization. SR and VD: methodology. SR, TK, and TB: formal analysis. SR: statistical analysis. SR and TK: writing—original draft preparation. SR, TK, TB, MT, and VD: writing—review and editing. SR and TB: visualization. MT and VD: supervision. VD: funding acquisition. All authors have read and agreed to the published version of the manuscript.

## Conflict of Interest

The authors declare that the research was conducted in the absence of any commercial or financial relationships that could be construed as a potential conflict of interest.

## Publisher’s Note

All claims expressed in this article are solely those of the authors and do not necessarily represent those of their affiliated organizations, or those of the publisher, the editors and the reviewers. Any product that may be evaluated in this article, or claim that may be made by its manufacturer, is not guaranteed or endorsed by the publisher.

## Abbreviations

VDR: vitamin D receptor
PD: Parkinson’s disease
GDNF: glial cell line-derived neurotrophic factor
MDS-UPDRS: MDS-Unified Parkinson’s Disease Rating Scale
H&Y: Hoehn and Yahr
SNP: single nucleotide polymorphism
AE: adverse event
DA: dopamine agonist
LED: levodopa equivalent dose
MFs: motor fluctuations
VHs: visual hallucinations
ICDs: impulse control disorders
ORs: odds ratios
CIs: confidence intervals.
