# Supplementary material for: Genetic Variability of the Vitamin D Receptor Affects Susceptibility to Parkinson’s Disease and Dopaminergic Treatment Adverse Events
Source: Front Aging Neurosci. 2022 Apr 19;14:853277. doi: 10.3389/fnagi.2022.853277 (PMC9063754; doi:10.3389/fnagi.2022.853277)
Supplement: Supplementary file 1 [file Data_Sheet_1.docx]

**Supplementary table 1: Power calculations**

| ***VDR* SNPs** | **PD risk** | **Motor fluctuations** | **Dyskinesia** | **EDS and sleep attacks** | **Visual hallucinations** |
| --- | --- | --- | --- | --- | --- |
| **rs739837** | <0.545, >1.828 | <0.447, >2.197 | <0.443, >2.211 | <0.426, >2.291 | <0.382, >2.531 |
| **rs4516035** | <0.543, >1.824 | <0.447, >2.198 | <0.443, >2.212 | <0.426, >2.292 | <0.383, >2.533 |
| **rs11568820** | <0.382, >2.063 | <0.449, >2.494 | <0.237, >2.490 | <0.207, >2.559 | <0.138, >2.780 |
| **rs731236** | <0.529, >1.822 | <0.424, >2.181 | <0.419, >2.190 | <0.401, >2.261 | <0.353, >2.477 |
| **rs2228570** | <0.518, >1.830 | <0.426, >2.180 | <0.422, >2.190 | <0.403, >2.262 | <0.355, >2.479 |
| **rs1544410** | <0.532, >1.820 | <0.429, >2.180 | <0.424, >2.211 | <0.405, >2.263 | <0.358, >2.481 |
| ***VDR* SNPs** | **Nausea/vomiting** | **Orthostatic hypotension** | **Peripheral edema** | **Impulse control disorders** |  |
| **rs739837** | <0.410, >2.373 | <0.433, >2.259 | <0.341, >2.808 | <0.283, >3.319 |  |
| **rs4516035** | <0.410, >2.375 | <0.433, >2.260 | <0.342, >2.812 | <0.284, >3.325 |  |
| **rs11568820** | <0.181, >2.635 | <0.218, >2.530 | <0.077, >3.028 | >3.451 |  |
| **rs731236** | <0.383, >2.336 | <0.408, >2.233 | <0.308, >2.722 | <0.245, >3.160 |  |
| **rs2228570** | <0.385, >2.337 | <0.410, >2.233 | <0.311, >2.725 | <0.248, >3.167 |  |
| **rs1544410** | <0.388, >2.338 | <0.413, >2.233 | <0.314, >2.730 | <0.252, >3.176 |  |

Numbers in the table represent ORs that can be detected with 80% certainty if the difference between groups really exists.

**Supplementary table 2: Clinical characteristics of the patient and control cohort**

| **Characteristic** | | **Patient cohort (N = 231)** | **Control cohort (N = 161)** |
| --- | --- | --- | --- |
| Gender | Male (%) | 132 (57.1) | 125 (77.6) |
|  | Female (%) | 99 (42.9) | 36 (22.4) |
| Side of disease initiation | Left (%) | 91 (39.4) |  |
|  | Both (%) | 21 (9.1) |  |
|  | Right (%) | 119 (51.5) |  |
| Tremor-predominant PD | No (%) | 46 (19.9) |  |
|  | Yes (%) | 185 (81.1) |  |
| Ever being treated with DAs** | No (%) | 57 (25.1) |  |
|  | Yes (%) | 170 (74.9) |  |
| Age at enrolment | Median (25%-75%), years | 72.5 (65.7–78.0) | 55 (52-58.5) |
| Age at diagnosis | Median (25%-75%), years | 62.1 (54.8 – 71.7) |  |
| Disease duration | Median (25%-75%), years | 7.6 (3.8 – 13.6) |  |
| Dopaminergic treatment duration*** | Median (25%-75%), years | 7.3 (3.6-13.5) |  |
| Levodopa treatment duration** | Median (25%-75%), years | 6.2 (2.4 – 11.2) |  |
| LED at enrolment*^,^** | Median (25%-75%), mg/day | 975 (600 – 1363.5) |  |
| **Adverse event** | **Number (%) of patients experiencing the adverse event** | | |
| Motor fluctuations | 123 (53.2) | | |
| Dyskinesia | 101 (43.7) | | |
| Excessive daytime sleepiness and sleep attacks | 81 (35,1) | | |
| Visual hallucinations**** | 57 (24,7) | | |
| Nausea/vomiting**** | 70 (30,3) | | |
| Orthostatic hypotension**** | 87 (37,7) | | |
| Peripheral oedema**** | 44 (19,0) | | |
| Impulse control disorders**** | 32 (13,9) | | |

*LED calculated according to Tomlinson et al.

**Data missing for four patients.

***Data missing for three patients.

****Data missing for one patient.

**Supplementary table 3: Influence of genetic variability on the severity of Parkinson's disease**

| **Single nucleotide polymorphism** | **Genotype** | **p-value** | **Median LED** |
| --- | --- | --- | --- |
| rs739837 | TT vs. TG+GG | 0.632 | 1040.0 vs. 932.5 |
|  | TT vs. TG vs. GG | 0.243 | 1040.0 vs. 975.0 vs. 831.25 |
| **rs4516035** | **TT vs. TC+CC** | **0.023** | **798.0 vs. 1029.75** |
|  | TT vs. TC vs. CC | 0.075 | 798.0 vs. 1047.8 vs. 1000.0 |
| rs11568820 | GG vs. GA+AA | 0.789 | 1000.0 vs. 900.0 |
|  | GG vs. GA vs. AA | 0.664 | 1000.0 vs. 840.0 vs. 1152.75 |
| rs731236 | TT vs. TC+CC | 0.408 | 905.0 vs. 1019.5 |
|  | TT vs. TC vs. CC | 0.420 | 905.0 vs. 1000.0 vs. 1200.0 |
| rs2228570 | CC vs. TC+TT | 0.246 | 958.0 vs. 998.75 |
|  | CC vs. TC vs. TT | 0.496 | 975.0 vs. 1000.0 vs. 958.0 |
| rs1544410 | GG vs. GA+AA | 0.569 | 930.0 vs. 1000.0 |
|  | GG vs. GA vs. AA | 0.380 | 930.0 vs. 962.5 vs. 1200.0 |

Statistically significant results are printed in bold text.

**Supplementary table 4: Excessive daytime sleepiness and sleep attacks and their association with *VDR* polymorphisms**

| **Gene** | **SNPs** | **Genotypes** | **Excessive daytime sleepiness and sleep attacks** | | |
| --- | --- | --- | --- | --- | --- |
|  |  |  | **OR** | **95% CI** | **p-value** |
| ***VDR*** | rs739837 | TT | Ref. |  |  |
|  |  | TG | 1.07 | 0.56 - 2.03 | 0.840 |
|  |  | GG | 1.21 | 0.57 - 2.58 | 0.618 |
|  |  | TG+GG | 1.11 | 0.61 - 2.03 | 0.727 |
|  | rs4516035 | TT | Ref. |  |  |
|  |  | CT | 1.56 | 0.81-2.99 | 0.185 |
|  |  | CC | 0.97 | 0.43-2.16 | 0.931 |
|  |  | CT+CC | 1.36 | 0.73-2.53 | 0.339 |
|  | rs11568820 | GG | Ref. |  |  |
|  |  | GA | 0.94 | 0.51-1.72 | 0.835 |
|  |  | AA | 0.60 | 0.12-3.05 | 0.535 |
|  |  | GA+AA | 0.90 | 0.50-1.61 | 0.711 |
|  | rs731236 | TT | Ref. |  |  |
|  |  | TC | 0.76 | 0.42-1.37 | 0.363 |
|  |  | CC | 1.00 | 0.44-2.28 | 0.989 |
|  |  | TC+CC | 0.81 | 0.47-1.42 | 0.466 |
|  | rs2228570 | CC | Ref. |  |  |
|  |  | TC | 1.72 | 0.93-3.18 | 0.084 |
|  |  | TT | 1.89 | 0.87-4.12 | 0.109 |
|  |  | TC+TT | 0.72 | 0.36-1.43 | 0.345 |
|  | rs1544410 | GG | Ref. |  |  |
|  |  | GA | 0.78 | 0.43-1.42 | 0.413 |
|  |  | AA | 0.87 | 0.38-2.02 | 0.750 |
|  |  | GA+AA | 0.80 | 0.45-1.41 | 0.440 |

**Supplementary table 5: Visual hallucinations and their association with *VDR* polymorphisms**

| **Gene** | **SNPs** | **Genotypes** | **Visual hallucinations** | | |
| --- | --- | --- | --- | --- | --- |
|  |  |  | **OR** | **95% CI** | **p-value** |
| ***VDR*** | rs739837 | TT | Ref. |  |  |
|  |  | TG | 0.82 | 0.40-1.69 | 0.585 |
|  |  | GG | 1.61 | 0.72-3.58 | 0.585 |
|  |  | TG+GG | 1.04 | 0.54-2.03 | 0.904 |
|  | rs4516035 | TT | Ref. |  |  |
|  |  | CT | 1.23 | 0.61-2.50 | 0.565 |
|  |  | CC | 0.67 | 0.27-1.70 | 0.399 |
|  |  | CT+CC | 1.04 | 0.53-2.06 | 0.900 |
|  | rs11568820 | GG | Ref. |  |  |
|  |  | GA | 1.14 | 0.59-2.22 | 0.695 |
|  |  | AA | 1.05 | 0.20-5.43 | 0.951 |
|  |  | GA+AA | 1.13 | 0.60-2.14 | 0.703 |
|  | rs731236 | TT | Ref. |  |  |
|  |  | TC | 0.55 | 0.29-1.04 | 0.067 |
|  |  | CC | 0.55 | 0.21-1.41 | 0.213 |
|  |  | TC+CC | 0.55 | 0.30-1.00 | 0.051 |
|  | rs2228570 | CC | Ref. |  |  |
|  |  | TC | 0.94 | 0.48-1.85 | 0.859 |
|  |  | TT | 1.48 | 0.65-3.36 | 0.348 |
|  |  | TC+TT | 0.65 | 0.31-1.37 | 0.260 |
|  | rs1544410 | GG | Ref. |  |  |
|  |  | GA | **0.43** | **0.22-0.84** | **0.013** |
|  |  | AA | 0.58 | 0.23-1.46 | 0.247 |
|  |  | GA+AA | **0.46** | **0.25-0.86** | **0.014** |

Statistically significant results are printed in bold text.

**Supplementary table 6: Nausea/vomiting and their association with *VDR* polymorphisms**

| **Gene** | **SNPs** | **Genotypes** | **Nausea/vomiting** | | |
| --- | --- | --- | --- | --- | --- |
|  |  |  | **OR** | **95% CI** | **p-value** |
| ***VDR*** | rs739837 | TT | Ref. |  |  |
|  |  | TG | 0.53 | 0.27-1.02 | 0.057 |
|  |  | GG | 0.84 | 0.40-1.80 | 0.659 |
|  |  | TG+GG | 0.62 | 0.34-1.14 | 0.121 |
|  | rs4516035 | TT | Ref. |  |  |
|  |  | CT | 0.94 | 0.49-1.80 | 0.850 |
|  |  | CC | 0.54 | 0.23-1.26 | 0.151 |
|  |  | CT+CC | 0.80 | 0.43-1.50 | 0.492 |
|  | rs11568820 | GG | Ref. |  |  |
|  |  | GA | 1.04 | 0.56-1.95 | 0.899 |
|  |  | AA | 0.76 | 0.15-3.92 | 0.747 |
|  |  | GA+AA | 1.01 | 0.55-1.85 | 0.979 |
|  | rs731236 | TT | Ref. |  |  |
|  |  | TC | 0.85 | 0.46-1.59 | 0.618 |
|  |  | CC | 1.38 | 0.60-3.17 | 0.447 |
|  |  | TC+CC | 0.96 | 0.54-1.72 | 0.897 |
|  | rs2228570 | CC | Ref. |  |  |
|  |  | TC | 0.87 | 0.46-1.63 | 0.655 |
|  |  | TT | 1.30 | 0.60-2.85 | 0.506 |
|  |  | TC+TT | 0.71 | 0.35-1.45 | 0.346 |
|  | rs1544410 | GG | Ref. |  |  |
|  |  | GA | 0.97 | 0.52-1.82 | 0.919 |
|  |  | AA | 1.48 | 0.64-3.45 | 0.363 |
|  |  | GA+AA | 1.07 | 0.59-1.94 | 0.823 |

**Supplementary table 7: Orthostatic hypotension and its association with *VDR* polymorphisms**

| **Gene** | **SNPs** | **Genotypes** | **Orthostatic hypotension** | | |
| --- | --- | --- | --- | --- | --- |
|  |  |  | **OR** | **95% CI** | **p-value** |
| ***VDR*** | rs739837 | TT | Ref. |  |  |
|  |  | TG | 1.16 | 0.61-2.22 | 0.652 |
|  |  | GG | **2.23** | **1.05-4.70** | **0.036** |
|  |  | TG+GG | 1.44 | 0.79-2.64 | 0.234 |
|  | rs4516035 | TT | Ref. |  |  |
|  |  | CT | 2.28 | 0.62-2.23 | 0.615 |
|  |  | CC | 1.08 | 0.50-2.33 | 0.846 |
|  |  | CT+CC | 1.15 | 0.63-2.10 | 0.656 |
|  | rs11568820 | GG | Ref. |  |  |
|  |  | GA | 1.25 | 0.69-2.26 | 0.469 |
|  |  | AA | 3.04 | 0.70-13.18 | 0.138 |
|  |  | GA+AA | 1.38 | 0.78-2.43 | 0.270 |
|  | rs731236 | TT | Ref. |  |  |
|  |  | TC | **0.52** | **0.29-0.93** | **0.026** |
|  |  | CC | **0.38** | **0.16-0.91** | **0.029** |
|  |  | TC+CC | **0.48** | **0.28-0.84** | **0.010** |
|  | rs2228570 | CC | Ref. |  |  |
|  |  | TC | 0.88 | 0.49-1.60 | 0.684 |
|  |  | TT | 1.44 | 0.68-3.05 | 0.341 |
|  |  | TC+TT | 0.65 | 0.33-1.29 | 0.217 |
|  | rs1544410 | GG | Ref. |  |  |
|  |  | GA | **0.48** | **0.26-0.86** | **0.013** |
|  |  | AA | **0.42** | **0.18-0.99** | **0.046** |
|  |  | GA+AA | **0.46** | **0.26-0.81** | **0.007** |

Statistically significant results are printed in bold text.

**Supplementary table 8: Peripheral oedema and their association with *VDR* polymorphisms**

| **Gene** | **SNPs** | **Genotypes** | **Peripheral oedema** | | |
| --- | --- | --- | --- | --- | --- |
|  |  |  | **OR** | **95% CI** | **p-value** |
| ***VDR*** | rs739837 | TT | Ref. |  |  |
|  |  | TG | 1.03 | 0.56-2.33 | 0.938 |
|  |  | GG | 1.80 | 0.74-4.37 | 0.198 |
|  |  | TG+GG | 1.26 | 0.59-2.67 | 0.547 |
|  | rs4516035 | TT | Ref. |  |  |
|  |  | CT | 1.44 | 0.62-3.34 | 0.395 |
|  |  | CC | 1.81 | 0.70-4.72 | 0.224 |
|  |  | CT+CC | 1.55 | 0.70-3.45 | 0.282 |
|  | rs11568820 | GG | Ref. |  |  |
|  |  | GA | 1.09 | 0.53-2.25 | 0.821 |
|  |  | AA | 0.61 | 0.07-5.14 | 0.649 |
|  |  | GA+AA | 1.03 | 0.51-2.09 | 0.935 |
|  | rs731236 | TT | Ref. |  |  |
|  |  | TC | **0.43** | **0.20-0.90** | **0.026** |
|  |  | CC | 1.08 | 0.44-2.68 | 0.868 |
|  |  | TC+CC | 0.56 | 0.29-1.09 | 0.088 |
|  | rs2228570 | CC | Ref. |  |  |
|  |  | TC | 1.51 | 0.71-3.24 | 0.284 |
|  |  | TT | 1.86 | 0.74-4.69 | 0.188 |
|  |  | TC+TT | 0.68 | 0.30-1.52 | 0.347 |
|  | rs1544410 | GG | Ref. |  |  |
|  |  | GA | 0.49 | 0.23-1.02 | 0.056 |
|  |  | AA | 1.12 | 0.45-2.81 | 0.812 |
|  |  | GA+AA | 0.61 | 0.31-1.20 | 0.151 |

Statistically significant results are printed in bold text.

**Supplementary table 9: Impulse control disorders and their association with *VDR* polymorphisms**

| **Gene** | **SNPs** | **Genotypes** | **Impulse control disorders** | | |
| --- | --- | --- | --- | --- | --- |
|  |  |  | **OR** | **95% CI** | **p-value** |
| ***VDR*** | rs739837 | TT | Ref. |  |  |
|  |  | TG | 1.22 | 0.49-3.03 | 0.667 |
|  |  | GG | 1.29 | 0.45-3.70 | 0.637 |
|  |  | TG+GG | 1.24 | 0.53-2.93 | 0.619 |
|  | rs4516035 | TT | Ref. |  |  |
|  |  | CT | 1.23 | 0.50-3.01 | 0.654 |
|  |  | CC | 0.90 | 0.29-2.79 | 0.855 |
|  |  | CT+CC | 1.13 | 0.48-2.66 | 0.788 |
|  | rs11568820 | GG | Ref. |  |  |
|  |  | GA | 0.96 | 0.42-2.21 | 0.924 |
|  |  | AA | / | / | / |
|  |  | GA+AA | 0.84 | 0.37-1.92 | 0.676 |
|  | rs731236 | TT | Ref. |  |  |
|  |  | TC | 1.81 | 0.78-4.21 | 0.167 |
|  |  | CC | 0.81 | 0.21-3.18 | 0.759 |
|  |  | TC+CC | 1.56 | 0.69-3.55 | 0.290 |
|  | rs2228570 | CC | Ref. |  |  |
|  |  | TC | 1.00 | 0.46-2.18 | 0.990 |
|  |  | TT | 0.27 | 0.06-1.25 | 0.095 |
|  |  | TC+TT | 3.68 | 0.84-16.06 | 0.083 |
|  | rs1544410 | GG | Ref. |  |  |
|  |  | GA | 1.57 | 0.67-3.64 | 0.299 |
|  |  | AA | 0.74 | 0.19-2.93 | 0.670 |
|  |  | GA+AA | 1.37 | 0.60-3.12 | 0.457 |

**Supplementary table 10: Motor fluctuations and their association with *VDR* polymorphisms**

| **Gene** | **SNPs** | **Genotypes** | **Motor fluctuations** | | |
| --- | --- | --- | --- | --- | --- |
|  |  |  | **OR** | **95% CI** | **p-value** |
| ***VDR*** | rs739837 | TT | Ref. |  |  |
|  |  | TG | 0.96 | 0.52-1.77 | 0.900 |
|  |  | GG | 0.87 | 0.42-1.79 | 0.696 |
|  |  | TG+GG | 0.93 | 0.52-1.65 | 0.802 |
|  | rs4516035 | TT | Ref. |  |  |
|  |  | CT | 0.87 | 0.47-1.60 | 0.650 |
|  |  | CC | 1.22 | 0.58-2.57 | 0.604 |
|  |  | CT+CC | 0.96 | 0.54-1.72 | 0.893 |
|  | rs11568820 | GG | Ref. |  |  |
|  |  | GA | 1.06 | 0.60-1.90 | 0.834 |
|  |  | AA | 6.57 | 0.79-54.67 | 0.081 |
|  |  | GA+AA | 1.24 | 0.71-2.18 | 0.449 |
|  | rs731236 | TT | Ref. |  |  |
|  |  | TC | 1.01 | 0.57-1.77 | 0.985 |
|  |  | CC | 1.75 | 0.77-3.98 | 0.184 |
|  |  | TC+CC | 1.14 | 0.67-1.95 | 0.636 |
|  | rs2228570 | CC | Ref. |  |  |
|  |  | TC | 1.25 | 0.70-2.22 | 0.453 |
|  |  | TT | 0.56 | 0.26-1.19 | 0.130 |
|  |  | TC+TT | **2.01** | **1.01-4.02** | **0.047** |
|  | rs1544410 | GG | Ref. |  |  |
|  |  | GA | 0.84 | 0.48-1.49 | 0.558 |
|  |  | AA | 1.57 | 0.68-3.61 | 0.287 |
|  |  | GA+AA | 0.96 | 0.56-1.67 | 0.896 |

Statistically significant results are printed in bold text.

**Supplementary table 11: Dyskinesia and their association with *VDR* polymorphisms**

| **Gene** | **SNPs** | **Genotypes** | **Dyskinesia** | | |
| --- | --- | --- | --- | --- | --- |
|  |  |  | **OR** | **95% CI** | **p-value** |
| ***VDR*** | rs739837 | TT | Ref. |  |  |
|  |  | TG | 1.30 | 0.70-2.40 | 0.407 |
|  |  | GG | 0.95 | 0.45-1.98 | 0.887 |
|  |  | TG+GG | 1.18 | 0.66-2.10 | 0.586 |
|  | rs4516035 | TT | Ref. |  |  |
|  |  | CT | 1.49 | 0.80-2.80 | 0.213 |
|  |  | CC | 1.55 | 0.73-3.28 | 0.256 |
|  |  | CT+CC | 1.51 | 0.83-2.74 | 0.177 |
|  | rs11568820 | GG | Ref. |  |  |
|  |  | GA | 0.71 | 0.39-1.28 | 0.253 |
|  |  | AA | 1.18 | 0.29-4.88 | 0.821 |
|  |  | GA+AA | 0.75 | 0.43-1.32 | 0.320 |
|  | rs731236 | TT | Ref. |  |  |
|  |  | TC | 1.11 | 0.63-1.97 | 0.718 |
|  |  | CC | 1.24 | 0.56-2.77 | 0.593 |
|  |  | TC+CC | 1.14 | 0.66-1.96 | 0.634 |
|  | rs2228570 | CC | Ref. |  |  |
|  |  | TC | 1.23 | 0.69-2.20 | 0.474 |
|  |  | TT | 1.13 | 0.54-2.39 | 0.748 |
|  |  | TC+TT | 0.99 | 0.50-1.96 | 0.980 |
|  | rs1544410 | GG | Ref. |  |  |
|  |  | GA | 0.97 | 0.55-1.73 | 0.919 |
|  |  | AA | 1.15 | 0.51-2.58 | 0.734 |
|  |  | GA+AA | 1.01 | 0.58-1.75 | 0.977 |
